# The epidemiology of migraine genetics: recent findings, implications, and future directions

**DOI:** 10.1186/1129-2377-14-S1-I1

**Published:** 2013-02-21

**Authors:** Tobias Kurth

**Affiliations:** 1 University of Bordeaux, Segalen, France

## 

There is little doubt about the strong contribution of genetic factors to migraine occurrence. However, while specific genes have been identified for the rare familiar hemiplegic migraine form, the contribution of genetic factors to more common forms of migraine on the population-level remains challenging. The complex heterogeneous clinical presentation, multifactorial triggering factors, and involvement of other disorders contribute to the difficulties identifying specific genetic variants for common migraine forms. Candidate gene association studies did not show convincing results and replication often failed. Recent collaborations of clinic- and population-based studies have identified several genetic variants in specific migraine subgroups but also on the population level. In addition, several studies have evaluated the role of genetic factors in the interrelationships of migraine and specific comorbidities. Some of the variants point towards involvement of biological reasonable mechanisms while the role of others remains unclear. This talk will summarize recent advances in migraine genetics in clinic- and population-based settings, discuss potential implications and pitfalls, as well as outlines future directions.

